# Health related quality of life in patients with chronic gastritis and peptic ulcer and factors with impact: a longitudinal study

**DOI:** 10.1186/1471-230X-14-149

**Published:** 2014-08-20

**Authors:** Zhengwei Wen, Xiaomei Li, Qian Lu, Julie Brunson, Miao Zhao, Jianfeng Tan, Chonghua Wan, Pingguang Lei

**Affiliations:** 1 People’s Hospital of Songgang, Baoan, Shenzhen, Guangdong 518105, China; 2 School of Public Health, Kunming Medical University, Kunming, Yunnan 650031, China; 3 Department of Psychology, University of Houston, Houston, TX 77204, USA; 4 Department of Psychology, Pennsylvania State University, Pennsylvania, PA, USA; 5 School of Humanities and Management, Research Center for Quality of Life and Applied Psychology, Guangdong Medical College, Dongguan 523808, China

**Keywords:** Chronic digestive disease, Health-related quality of life, SF-36, Influence factors, Standardized response mean

## Abstract

**Background:**

The assessment of Health Related Quality of Life (HRQOL) has been applied as a significant outcome indicator for patients with chronic diseases. No HRQOL study, however, has looked at HRQOL in patients with chronic gastritis and peptic ulcers. This paper focuses on comparing HRQOL in patients with chronic gastritis and peptic ulcers and examining the factors that influence the HRQOL of such patients. Results can be used for making decisions in clinical trials as well as aiding individual management and preventive care of these diseases.

**Methods:**

The Chinese version of the SF-36 (CSF-36) was administered twice to 244 patients with chronic gastritis and peptic ulcers. Mean scores across the two disease groups were compared using t-tests, change over time was analyzed with paired samples t-tests, and factors predicting HRQOL were investigated using the univariate general linear model.

**Results:**

The mean domain scores of patients with chronic gastritis were lower than those for patients with peptic ulcers, with the exception of physical functioning. Both groups had lower HRQOL compared with population norms. Mean domain scores increased after treatment in both groups. HRQOL in patients with these two chronic diseases differed by age, education level, marriage, income, and gender, but their explanatory power was relatively low.

**Conclusion:**

Quality of life of patients with chronic gastritis was lower than that of patients with peptic ulcers, which was lower than population norms. Quality of life in both patients groups was associated with socio-demographic risk factors.

## Background

Diseases of the digestive system such as gastritis, functional gastrointestinal disorders, and peptic ulcers are common throughout the world [1-3]. While these are not generally life-threatening conditions, they can significantly impair patients’ quality of life (QOL). These digestive system diseases are important to public health because they are remarkably common, can hinder a person’s daily activities, and can cause major social and economic burden. Peptic ulcer disease (PUD) affects as many as 10% of people in the United States at some point during their life, and the annual cost of treatment in the United States alone has been estimated at $5.7 billion [1]. Thus, there is a substantial impact of these diseases on public health.

In the last 30 years, health–related quality of life (HRQOL) has become an important outcome measure for patients with cancer and chronic diseases. The Medical Outcomes Study 36-items Short-Form Health Survey (SF-36) has been widely used as a HRQOL instrument [4,5] and is also being used with greater frequency internationally. The SF-36 has been translated into many foreign languages and is used in more than 40 countries as part of the International Quality of Life Assessment (IQOLA) project [5].

In China, research on HRQOL has sharply increased since the 1980s. The simplified Chinese (Mandarin) version of SF-36 (CSF-36) was rigorously developed using forward and backward translation and has demonstrated good reliability and validity in the Chinese general population [6]. More specifically, a three-stage protocol was followed including translation, tests of scaling construction and scoring assumptions, and validation and normalization. A total of 1688 respondents were recruited by multi-stage mixed sampling in 1000 households in 18 communities of Hangzhou. The results showed the clustering and ordering of item means was the same as that of the source survey. The item-scale correlations were identical for all except the social functioning and vitality scales; convergent validity and discriminant validity were satisfactory for all except the social functioning scale. In addition, Cronbach’s α coefficients ranged from 0.72 to 0.88, with the exception of 0.39 for the social functioning scale and 0.66 for the vitality scale, and two weeks test-retest reliability coefficients ranged from 0.66 to 0.94. The translated survey was also able to distinguish between known groups. It was concluded that the Chinese (mainland) version of the SF-36 functioned in the general population quite similarly to the original American population tested [6].

In addition, the CSF-36 was validated among patients with chronic diseases including hypertension, coronary heart disease, chronic gastritis and peptic ulcers in China [7]. In this longitudinal study, the CSF-36 was used in a sample of 534 patients from these four chronic disease groups, and the psychometric properties of the scale were evaluated by indicators such as Cronbach’s α, Pearson’s r, standardized response mean employing correlational analyses, multi-trait scaling analysis, t-tests, factor analyses, and structural equation models. It was concluded that the CSF-36 showed good validity and reliability but low responsiveness when used in patients, and it is a relatively useful instrument for patients with chronic disease when no specific instruments are available [7].

Worldwide, while there have been a few studies that used the SF-36 to measure HRQOL in patients with peptic ulcers [8-10], to date, there have been no studies which have compared HRQOL in patients with chronic gastritis and peptic ulcers using the SF-36. In a study conducted by Hallerbäck et al. [11], the Psychological General Well-Being Index (PGWB) was administrated to 1526 patients with esophagitis (192), gastric ulcers (109), duodenal ulcers (426), duodenitis/gastritis (296), negative endoscopy (401) and other diagnoses (102) before endoscopy. All five patient groups, excluding the other group, reported low general well-being compared to the general population; however, no differences emerged between the groups. On the other hand, in a study by Mokrowiecka et al. which used the SF-36, differences in HRQOL were found between patients with three common gastroenterological chronic conditions: gastroesophageal reflux disease (GERD), peptic ulcer disease (PUD) and ulcerative colitis (UC). Bodily pain measured by SF-36 was significantly lower in GERD and PUD than those with UC [8]. Therefore, we expect that SF-36 will allow the detection of different quality of life among patients with peptic ulcers and patients with chronic gastritis. We hope to fill a void in the literature by comparing these clinically similar diseases in an attempt to identify and explain differences in HRQOL outcomes. These results may be influential in making decisions in clinical trials as well as in aiding in the individual management of these diseases.

The goals of the present study are to (1) compare HRQOL using CSF-36 in patients with chronic gastritis and peptic ulcers, (2) compare HRQOL between both groups of patients and population norms, (3) determine the influence factors of HRQOL in these populations, and (4) compare HRQOL change over time and explore the factors that influence HRQOL differences after treatment.

## Methods

### Patients

Participants consisted of 244 inpatients with either chronic gastritis or peptic ulcers at the First Affiliated Hospital of Kunming Medical University. They were diagnosed primarily through endoscopy and gastric biopsy but also through gastrointestinal symptoms such as abdominal pain, bloating, and other symptoms of these two diseases. If any patients had both diseases, they were classified according to their primary diagnosis. All patients were considered for inclusion, but it was required that they were able to read, understand, and complete the questionnaires. Patients who were illiterate and those with advanced disease status were therefore excluded from participation.

### Measure

**Figure 1:**
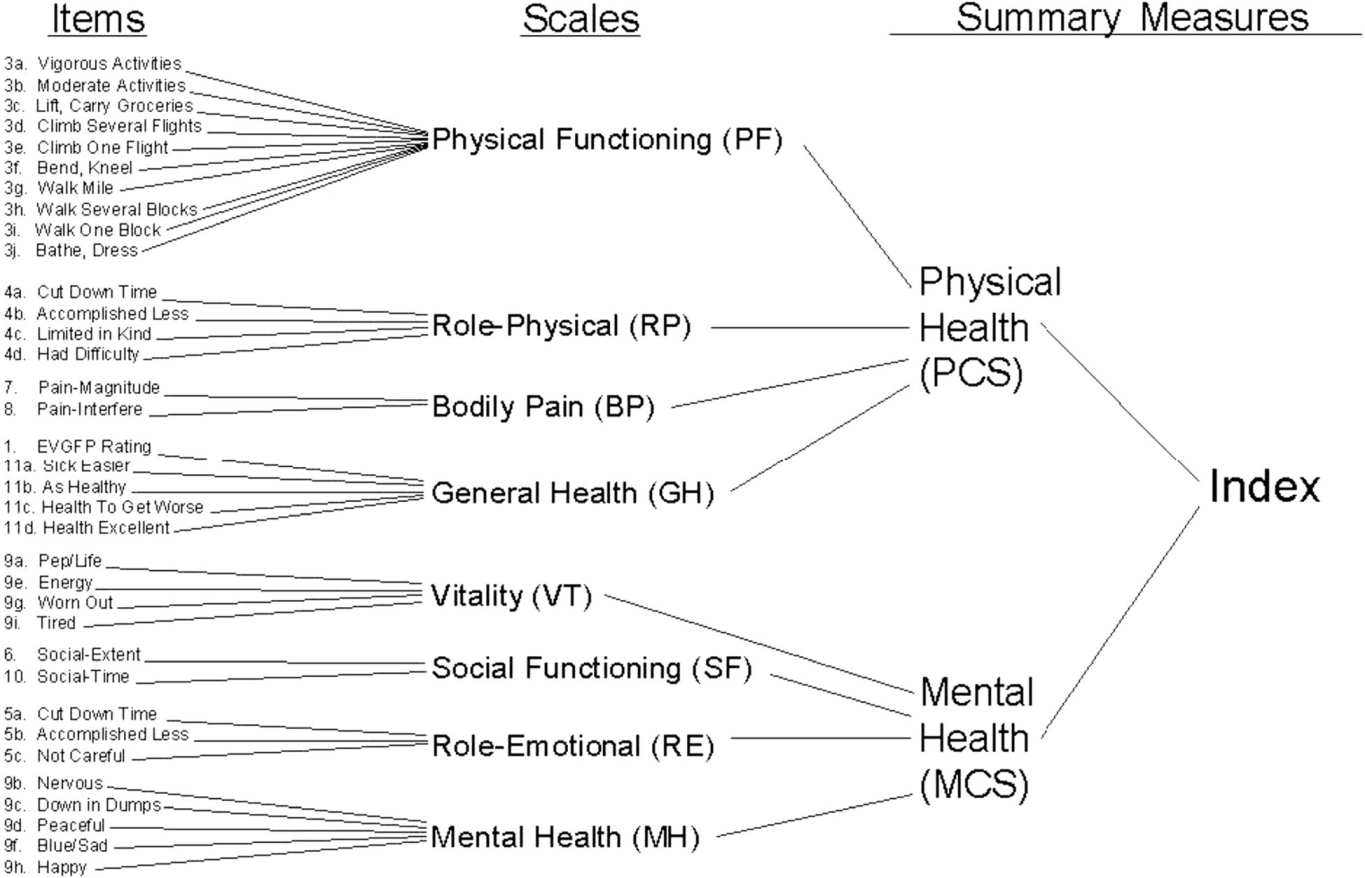
The conceptual structure of SF-36 (From Ware JE Jr et al.).

The CSF-36 contains 36 items which represent eight domains: physical functioning (PF), role-physical (RP), bodily pain (BP), general health (GH), vitality (VT), social functioning (SF), role-emotional (RE), and mental health (MH). These domains can be further aggregated into a physical components summary (PCS) and a mental components summary (MCS), or an overall index if needed (see Figure 1) [12]. Specifically, all domain scores and the two component summaries are linearly converted into a 0–100 scale using the following formula: SS = (RS-Min) × 100/R, where SS, RS, Min, and R represent the standardized score, raw score, minimum score, and range of scores, respectively, with higher scores representing better HRQOL [12].

### Survey method

After obtaining appropriate institutional review board approval of Kunming Medical University, the investigators described the study and the scale to the patients and obtained informed consent from patients who agreed to participate and met the inclusion criteria. Participants completed the CSF-36 (paper format) at the time of admission and at the time of discharge. Information regarding socio-demographic and clinical variables such as age, gender, and education was collected through medical records and the face page of our questionnaire.

### Statistical analysis

Domain scores for each of the two diseases were compared using t-tests. Independent samples t-tests were used to compare the mean scores for each domain and summary variables of the CSF-36 at admission across the two disease groups. In addition, independent samples t-tests were used to compare differences in each domain between the two disease groups. The change of HRQOL scores from admission to discharge was evaluated in several different ways: with a paired sample t-test for each domain of the CSF-36 separately for each disease, with a change ratio (CR, the difference between the two divided by the baseline means), and with a standardized response mean (SRM) [13,14], used to reflect the degree of change. Of them, SRM was used as a measure of effect size, with values of 0.20, 0.50 and 0.80 representing small, moderate and large responsiveness respectively [13,14].

In order to investigate what socio-demographic and disease type factors may influence HRQOL, the univariate general linear model was used with the information gathered at the time of admission (due to the larger sample size). PCS and MCS scores were used as the dependent variable respectively (backward selection with p-value in = 0.05 and p-value out = 0.10), and age as the covariate. The categorical independent variables were recoded before analysis as follows: gender (1 = male, 2 = female), marriage (1 = married, 2 = other), education (1 = primary school, 2 = high school, 3 = college or higher), ethnicity (1 = Han, 2 = other), medical security (1 = self-pay or partly-pay, 2 = public insurance), occupation (1 = factory worker, 2 = other occupations), disease type (1 = chronic gastritis, 2 = peptic ulcer). Finally, the univariate general linear model was used to analyze HRQOL score change from admission to discharge with the PCS and MCS score change as the dependent variable respectively and factors above as independent variables.

## Results

### Socio-demographic characteristics of the study population

Table 1 summarizes the socio-demographic variables of interest in the current sample. Of the 244 patients who completed measures at admission, 124 had been diagnosed with chronic gastritis and 120 had been diagnosed with peptic ulcers. Ages ranged 16 to 79, with a median age of 44.5 and mean (±SD) age of 45.2 (±15.4). A majority of the patients were of Han ethnicity (86.9%), male (58.2%) and married (85.2%). Forty-five patients (18.4%) had finished primary school, while 119 (48.8%) had completed high school, and 80 (32.8%) had a college degree. Of those who completed the admission measures, 84.4% (206) also completed the measures at discharge (chronic gastritis, peptic ulcers).

**Table 1:** Socio-demographic characteristics of the sample

**Characteristics**	**Chronic gastritis (N = 124)**		**Peptic ulcer (N = 120)**		**Total (N = 244)**	
	**n**	**%**	**n**	**%**	**n**	**%**
**Gender**						
Male	60	48.4	82	68.3	142	58.2
Female	64	51.6	38	31.7	102	41.8
**Marital status**						
Married	103	83.1	105	87.5	208	85.2
Other	21	16.9	15	12.5	36	14.8
**Age**						
<30	28	22.6	18	15	46	18.9
30-39	32	25.8	19	15.8	51	20.9
40-49	17	13.7	27	22.5	44	18.0
50-59	23	18.5	32	26.7	55	22.5
≥60	24	19.4	24	20.0	48	19.7
$\bar{X}\pm S$	43.7 ± 15.6		46.7 ± 15.2			
**Ethnicity**						
Han	108	87.1	104	86.7	212	86.9
Other	16	12.9	16	13.3	32	13.1
**Education**						
Primary school	24	19.4	21	17.5	45	18.4
High school	57	46.0	62	51.7	119	48.8
College/higher	43	34.7	37	30.8	80	32.8
**Occupation**						
Worker	36	29.0	46	38.3	82	33.6
Farmer	14	11.3	14	11.7	28	11.5
Teacher	10	8.1	14	11.7	24	9.8
Office-bearer	25	20.2	18	15	43	17.6
Others	39	31.5	28	23.3	67	27.5
**Perceived income**						
Poor	25	20.2	35	29.2	60	24.6
Fair	87	70.2	71	59.2	158	64.8
High	12	9.7	14	11.7	26	10.7
**Medical security**						
Self-paid	51	41.1	41	34.2	92	37.7
Partly-pay	7	5.6	6	5	13	5.3
Public insurance	66	53.2	73	60.8	139	57.0

### HRQOL comparisons across diseases

**Figure 2:**
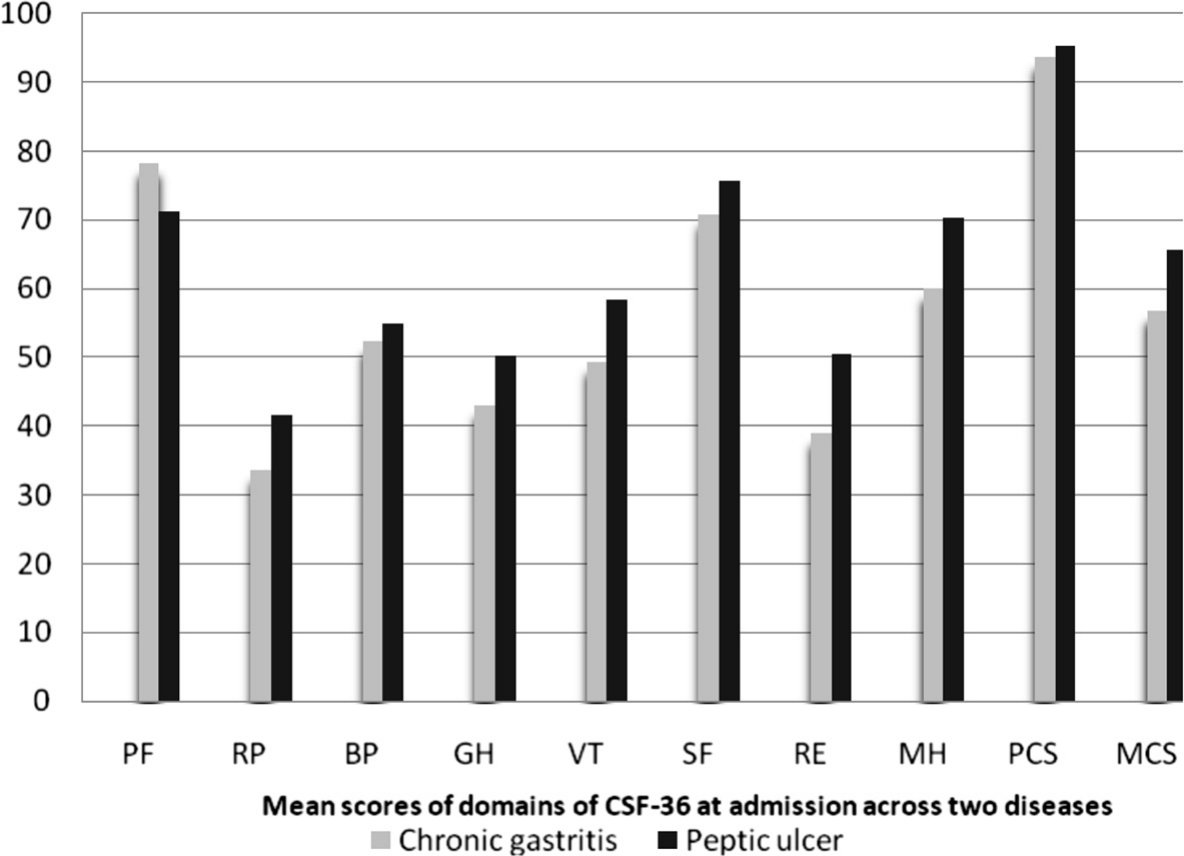
Mean scores of domains of CFS-36 at admission across two diseases.

At admission, the data from 244 patients (124 chronic gastritis and 120 peptic ulcers) were used to analyze HRQOL across diseases. As shown in Table 2 and Figure 2, most domains (with the exception of RP, BP, SF and PCS) exhibited statistically significance differences between the two disease groups. The patients with peptic ulcers had higher mean scores than those with chronic gastritis for most domains, with the exception of PF (where patients with chronic gastritis had higher scores).

**Table 2:** **Comparisons of mean scores (**$\bar{X}\pm S$**) of domains of CSF-36 across two diseases at admission and discharge**

**Domains**	**At admission (n = 244)**			**At discharge (n = 206)**		
	**Chronic gastritis**	**Peptic ulcer**	***t***	**Chronic gastritis**	**Peptic ulcer**	***t***
	**(n = 124)**	**(n = 120)**		**(n = 104)**	**(n = 102)**	
**PF**	78.25 ± 22.97	71.22 ± 28.91	2.10*	87.65 ± 16.71	83.81 ± 16.63	1.64
**RP**	33.67 ± 39.69	41.60 ± 41.84	-1.52	30.77 ± 38.51	24.75 ± 34.91	1.17
**BP**	52.27 ± 20.86	54.93 ± 24.26	-0.92	51.92 ± 20.02	52.65 ± 22.56	-0.24
**GH**	43.06 ± 19.67	50.29 ± 19.87	-2.86*	51.25 ± 18.71	55.59 ± 18.05	-1.69
**VT**	49.40 ± 21.10	58.29 ± 21.79	-3.24*	55.14 ± 19.31	62.28 ± 15.84	-2.89*
**SF**	70.79 ± 23.52	75.56 ± 21.44	-1.65	74.15 ± 21.70	75.93 ± 18.64	-0.63
**RE**	38.98 ± 41.80	50.42 ± 40.91	-2.16*	46.15 ± 41.88	44.77 ± 40.76	0.24
**MH**	60.03 ± 20.39	70.30 ± 17.40	-4.22*	67.73 ± 18.05	73.69 ± 14.05	-2.64*
**PCS**	93.67 ± 27.10	95.22 ± 31.56	-0.41	103.82 ± 24.09	103.35 ± 21.62	0.14
**MCS**	56.89 ± 18.51	65.66 ± 15.65	-3.98*	63.19 ± 17.07	68.66 ± 12.05	-2.65*

PF: physical function, RP: role-physical, BP: bodily pain, GH: general health, VT: vitality, SF: social function, RE: role-emotional, MH: mental-health, PCS: Physical Component Summary, MCS: Mental Component Summary.

*p < 0.05.

In addition, based on the data from 206 patients (104 chronic gastritis and 102 peptic ulcers), mean scores for each domain and summary variable of the CSF-36 at discharge were compared (see Table 2). Only VT, MH, and MCS were significantly different between the two groups, with scores for patients with peptic ulcers being higher than those of patients with chronic gastritis.

### HRQOL comparisons between patients and population norms

Work conducted in China with a community-based sample of 1688 showed mean domain scores of PF 82.2 ± 19.8, RP 81.2 ± 33.6, BP 81.5 ± 20.5, GH 56.7 ± 20.2, VT 52.0 ± 20.9, SF 83.0 ± 17.8, RE 84.4 ± 32.4, and MH 59.7 ± 22.7 [6]. When compared to this normal sample, our sample showed (at admission) lower mean scores in seven domains for both patients with chronic gastritis and patients with peptic ulcers (p < 0.001); there was no significant difference in MH scores for those with chronic gastritis and higher MH scores than that of the population norm for those with peptic ulcers.

### HRQOL change after treatments

The mean duration of time between pre-and post-treatment assessments was 8.03 ± 3.85 days for chronic gastritis, and 7.11 ± 2.86 days for peptic ulcer. As shown in Table 3, the mean scores for most domains and for the two component summaries were significantly different before and after treatment (with the exceptions of BP and RE for chronic gastritis, and BP, SF, and RE for peptic ulcers). Overall, HRQOL changed more for patients with peptic ulcers than for patients with chronic gastritis, with most domains showing an increase except for RP in both patients with chronic gastritis and peptic ulcers. However, the only domains that were significantly different between the two disease groups were RP, RE, and MH (p < 0.05). For patients with chronic gastritis, the domains of PF, GH, and MH, and also the PCS and MCS, had higher SRM (>0.60) after treatment, indicating a moderate effect size. A possible reason for this is that these domains are largely affected by pain, and the pain was reduced after treatment.

**Table 3:** CSF-36 domain score change after treatment for each disease

**Domains**	**Chronic gastritis**			**Peptic ulcer**		
	**DIF (**$\bar{X}\pm S$**)**	CR (%)	SRM	**DIF (**$\bar{X}\pm S$**)**	CR (%)	SRM
Physical functioning (PF)	10.10 ± 15.80	12.93	0.64*	11.25 ± 23.47	15.53	0.48*
Role-physical (RP)	-5.29 ± 26.91#	-14.67	0.20*	-15.75 ± 38.21#	-38.89	0.41*
Bodily pain (BP)	-0.63 ± 15.90	-1.21	0.04	1.82 ± 19.76	-3.36	0.09
General health (GH)	7.26 ± 11.98	16.50	0.61*	5.69 ± 13.33	11.39	0.43*
Vitality (VT)	4.23 ± 9.57	8.31	0.44*	3.66 ± 15.94	6.25	0.23*
Social functioning (SF)	3.53 ± 15.03	4.99	0.23*	-0.22 ± 14.24	-0.29	0.02
Role-emotional (RE)	3.21 ± 30.29#	7.46	0.11	-6.27 ± 37.63#	-12.26	0.17
Mental health (MH)	7.12 ± 11.37#	11.74	0.63*	3.61 ± 12.52#	5.15	0.29*
**PCS**	9.61 ± 14.63	10.13	0.66*	8.05 ± 21.38	8.46	0.38*
**MCS**	5.33 ± 8.69	9.21	0.61*	2.93 ± 10.10	4.46	0.29*

**PCS**: Physical Component Summary (weighted sum of PF, RP, BP and GH).

**MCS**: Mental Component Summary (weighted sum of VT, SF, RE and MH).

**DIF**: Difference between admission and discharge (scores at discharge minus scores at admission).

*p < 0.05 by paired t-test.

#p < 0.05 by independent samples t-test.

### Factors with impact on HRQOL

Results suggest that different domains of HRQOL are influenced by different socio-demographic variables as well as disease type (see Table 4). Higher PCS scores tended to be associated with patients who were married, younger, and had a higher education and income level. Higher MCS scores were associated with being male, having a higher education and income level, and having a peptic ulcer. For example, the PCS score for the married patients was on average 11.95 units higher than that of the other patients (single, divorced, etc.); the PCS score for the patients with education level one (primary school) was on average 11.70 units lower than that of the patients at education level three (college or higher) as suggested by the parameter coefficient of -11.70. Similarly, the MCS score for male patients was on average 7.78 units higher than that of the female patients; the MCS score for the patients with education level one (primary school) was on average 5.85 units lower than that of the patients at education level three (college or higher). However, it is noted that the MCS score for the patients with chronic gastritis was on average 7.48 units lower than that of the patients with peptic ulcers, given the other factors remain stable. This implies that the effects of the socio-demographic factors on PCS are the same across the disease types, but the effects of the factors on MCS scores were different. In other words, the effects of the factors on MCS were dependent on disease type, but the effects of the factors on PCS were not dependent on disease type.

**Table 4:** Factors with impact on HRQOL in patients with chronic diseases selected by the univariate general linear models

**Domains**	**Factors**	**B**	**Std. error**	***t***	***P***
Physical Component Summary (PCS)	Constant	123.78	7.93	15.61	0.000
	Marriage =1 (married)	11.95	5.47	2.13	0.030
	**Marriage = 2 (other)**	**.**	**.**	**.**	**.**
	Education = 1 (primary school)	-11.70	5.35	-2.19	0.030
	Education = 2 (high school)	-1.63	4.05	-0.40	0.688
	**Education = 3 (college or higher)**	**.**	**.**	**.**	**.**
	Perceived income = 1 (poor)	-17.39	6.61	-2.63	0.009
	Perceived income = 2 (fair)	-7.56	5.93	-1.28	0.203
	**Perceived income = 3 (high)**	**.**	**.**	**.**	**.**
	Age	-0.60	0.13	-4.66	0.000
	Adjusted R Square = 0.14 F = 7.64, P = 0.000				
Mental Component Summary (MCS)	Constant	68.94	4.05	17.93	0.000
	Gender =1 (male)	7.78	2.20	3.53	0.000
	**Gender = 2 (female)**	**.**	**.**	**.**	**.**
	Education = 1 (primary school)	-5.85	3.01	-1.96	0.050
	Education = 2 (high school)	2.38	2.43	0.98	0.328
	**Education = 3 (college or higher)**	**.**	**.**	**.**	**.**
	Perceived income = 1 (poor)	-14.56	3.38	-3.79	0.000
	Perceived income = 2 (fair)	-7.66	3.45	-2.22	0.027
	**Perceived income = 3 (high)**	**.**	**.**	**.**	**.**
	Disease = 1 (chronic gastritis)	-7.48	2.14	-3.50	0.001
	**Disease = 2 (peptic ulcer)**	**.**	**.**	**.**	**.**
	Adjusted R Square = 0.17 F = 9.09, P = 0.000				

B: Regression coefficient, Std Error: Standard error of Coefficients B.

Categories in bold were as references.

The influence of the socio-demographic and disease type factors in predicting PCS and MCS score change after treatment were different from that above (see Table 5 in detail). Changes in PCS were influenced by marriage, age and occupation, whereas changes in MCS were only influenced by gender and marriage.

**Table 5:** Factors with impact on HRQOL change after treatments in patients with chronic diseases selected by the univariate general linear models

**Domains**	**Factors**	**B**	**Std. error**	***t***	***P***
Physical Component Summary (PCS)	Constant	6.44	4.19	1.54	0.126
	Marriage =1 (married)	-8.15	3.94	-2.07	0.040
	**Marriage = 2 (other)**	**.**	**.**	**.**	**.**
	Occupation =1 (worker)	4.71	2.69	1.75	0.080
	**Occupation =2 (other)**	**.**	**.**	**.**	**.**
	Age	0.17	0.09	1.82	0.070
	Adjusted R Square = 0.03 F = 2.92, P = 0.035				
Mental Component Summary (MCS)	Constant	9.96	1.83	5.45	0.000
	Gender =1 (male)	-2.54	1.30	-1.97	0.050
	**Gender = 2 (female)**	**.**	**.**	**.**	**.**
	Marriage =1 (married)	-5.09	1.80	-2.82	0.005
	**Marriage = 2 (other)**	**.**	**.**	**.**	**.**
	Adjusted R Square = 0.05, F = 5.89, P = 0.003				

B: Regression coefficient, Std Error: Standard error of Coefficients B.

Categories in bold were as references.

## Discussions

The role of patient-based symptoms and QOL assessment in evaluating and treating gastrointestinal disorders has gained increased attention among gastrointestinal researchers in recent years. We found that patients with chronic gastritis had lower quality of life compared with patients with peptic ulcers (except for PF domain), and both groups had lower quality of life compared with population norms (except for MH domain). Both groups experienced increased QOL after treatments (except for RP domain for two diseases). Patients who were female, with lower income, lower education, and older age tended to have lower quality of life.

Patients with peptic ulcers tended to be older than patients with chronic gastritis (with mean ages of 46.7 and 43.7, respectively), which may explain the differences between groups, as increases in age are associated with a decline in physical functioning and an increase in mental functioning [15,16]. Another explanation is that the differences may be due to gender; there were more women than men in the chronic gastritis group and more men than women in the peptic ulcer group. Additionally, patients with chronic gastritis tended to be in worse health than those with peptic ulcers when they were hospitalized. However, to understand the cause of this, further study is needed. Future research should further examine the differences in HRQOL between the two groups as well as explore possible explanations for the differences.

Past work has shown that patients with chronic disease tend to have lower mean domain scores than the general population, due to the symptoms and side effects caused by treatments [11,17,18]. For example, in Hallerbäck’s study [11], the results showed that all five patient groups with digestive diseases reported low general well-being compared with the general population. When compared to Chinese population norms [6], our sample also showed lower mean scores in seven domains for both patients with chronic gastritis and patients with peptic ulcers, (the only exception being MH), which supports these existing findings.

In general, patients with gastrointestinal disorders tend to have lower QOL than the general population, and treatment of the disorder can improve QOL [16-21]. For example, Glise investigated 392 patients with peptic ulcers and found that the general QOL score for the PGWB increased significantly during treatment up until two weeks post-treatment [21]. The change in domain scores from admission to discharge reflects not only the effect of treatment but also the internal responsiveness of the instrument [13,14]. Table 3 shows that most domain scores changed greatly after treatment for both those with chronic gastritis and peptic ulcers (moderate and large SRM). The increase in domain scores after treatment would suggest greater QOL, however for patients in both disease groups the mean score of role-physical declined. This decline may be explained by the nature of the instrument; 4 items (4a, 4b, 4c, 4d) probe role limitations that are a result of physical health (e.g., “Cut down on the amount of time you spent on work or other activities”). Patients who just spent time in the hospital being treated are likely to respond affirmatively to these items.

It is notable that some domains (e.g. bodily pain, role-emotional) had no significant differences after treatment. Some possible explanations for this are: (1) the observation period (approximately two weeks) may have been too short to observe significant changes; (2) some domains may not change over time; (3) insufficient sample size; and (4) the CSF-36 may not be sensitive enough for use in work examining chronic disease, as it was developed for the general population [22].

Lower HRQOL scores have previously been associated with lower socioeconomic status, educational attainment, and age [23-27]. Our results supported these findings, as HRQOL was related to age, education, marriage, and disease type, although different domains were influenced by different factors. Overall, patients’ education level and perceived income were the most influential factors, as those with a higher education level and with higher perceived income tended to have higher domain scores. The reason for this may be that the people with a higher education level and higher perceived income have better life conditions. HRQOL scores in patients living with a spouse were greater than those of patients without a spouse, which may be due to the additional support a patient may receive from a spouse versus other family members.

Patients with peptic ulcers tended to have higher scores on the mental component summary than those with chronic gastritis after controlling for other factors. There were also some observed gender effects, as male patients tended to have higher scores for the mental component summary after controlling for other factors, which is in accordance with other findings using the SF-36 [28,29]. Although systematic differences in HRQOL between men and women is a common finding, very few explanations or interpretations have been presented as to why this is the case. It may be that women experience greater, and/or different, stressors than the men. Ultimately, it is not clear why the effects of the socio-demographic factors on the mental component summary scores were dependent on disease type whereas the effects of the factors on physical component summary scores were not. Most likely, this is because there were differences in MCS scores across the two groups but no differences in PCS scores across the groups. Further research is needed to investigate the nature of these differences and their associated consequences. The factors influencing HRQOL also impacted longitudinal change after treatment. Changes in the physical component summary scores were influenced by marriage, age and occupation, whereas changes in the mental component summary scores were only influenced by gender and marriage. The reasons for these differences should be studied further. However, it should be noted that these factors had lower explanatory power for HRQOL as indicated by the relatively small adjusted R-squared (0.14 for PCS and 0.17 for MSC, 0.03 for PCS change and 0.05 for MCS change). We plan to investigate the effect of clinical variables such as disease stage, severity, and comorbidities in future studies.

It is important to note that the participants were only selected from a hospital and there was a fairly small sample size, which may be a limitation of the study. Additional community studies with larger sample sizes are needed. In addition, this study focused on HRQOL changes as indicated by statistical significance; however, clinically meaningful differences were not considered, which are more important than statistically significant differences. Our future directions include analyzing the minimal clinical important difference of QOL scores for these two diseases by anchor-based methods and distribution-based methods.

Despite these limitations, we found that patients with chronic gastritis had lower HRQOL than patients with peptic ulcers. Both groups had lower HRQOL at admission than the general population. In general, quality of life increased after treatment in both groups, and HRQOL in patients with these two chronic diseases differed by age, education level, marriage, income, and gender. Patients who were female, with lower income, lower education, and older age, tended to have a lower quality of life. Such information could be useful for making decisions in clinical trials as well as for individual management and rehabilitation of these diseases [30,31]. Well-executed HRQOL research such as this helps inform those tasked with health rationing or anyone involved in the decision-making process for agencies such as the Food and Drug Administration, European Medicines Agency or National Institute for Clinical Excellence [31].

## Conclusion

In conclusion, this is the first study to investigate quality of life and its association with socio-demographic risk factors among Chinese patients with peptic ulcers and chronic gastritis. Future studies should investigate why quality of life is linked to socio-demographic factors and clinical factors.

## Competing interests

The authors declare that they have no competing interests.

## Authors’ contributions

ZW, PL contributed to the design of the study, acquired and analyzed the data; XL contributed to the design of the study, analysis of the data and was involved in drafting the manuscript; QL contributed to the analysis of the data and revision of the manuscript; JB contributed to the analysis of the data and revision of the manuscript; MZ contributed to data-acquirement and analysis; JT contributed to data-acquirement and analysis; CW contributed to design of the study, data-acquirement and drafted the manuscript. All authors have read and approved the final manuscript.

## Authors’ information

# Zhengwei Wen and Xiaomei Li are as the first co-author with the same contributions.

## Pre-publication history

The pre-publication history for this paper can be accessed here:

http://www.biomedcentral.com/1471-230X/14/149/prepub
